# Acute coronary syndrome with non ST segment elevation myocardial infarction revealing anomalous connection of the left anterior descending artery

**DOI:** 10.11604/pamj.2019.32.138.17955

**Published:** 2019-03-25

**Authors:** Abdelmajid Bouzerda, Zouhair Lakhal, Laila Bendriss, Ali Khatouri

**Affiliations:** 1 Department of Cardiology, Mohammed V Military Hospital Hay Riad, Rabat, Morocco; 2 Department of Cardiology, Avicenne Military Hospital, Cadi Ayyad University, Marrakech, Morocco

**Keywords:** Congenital coronary artery abnormalities, anomalous connection, acute coronary syndrome

## Abstract

The occurrence of an acute coronary syndrome (ACS) with an anomalous connection of coronary artery (ANOCOR) identified as a culprit artery is very rare. This association may lead in some anatomical forms to a delay in coronary reperfusion. We report the clinical case of a patient admitted for high-risk Non ST elevation myocardial infarction (NSTEMI) in whom coronary angiography accidentally discovers an anomalous connection of the left coronary network from the right anterior. In light of this case and a review of literature, we discuss the peculiarities of these anomalous connections.

## Introduction

Anomalous connections of coronary arteries are a rare entity. To date, coronary angiography remains the reference exam. However, identifying the origin and path of aberrant coronary arteries by angiography may be difficult. From a case we have encountered in the cardiology department of Mohammed V Military hospital in Rabat, we propose to recall these coronary abnormalities, their clinical consequences and the performance of coronary angiography in their diagnosis.

## Patient and observation

**Figure 1:**
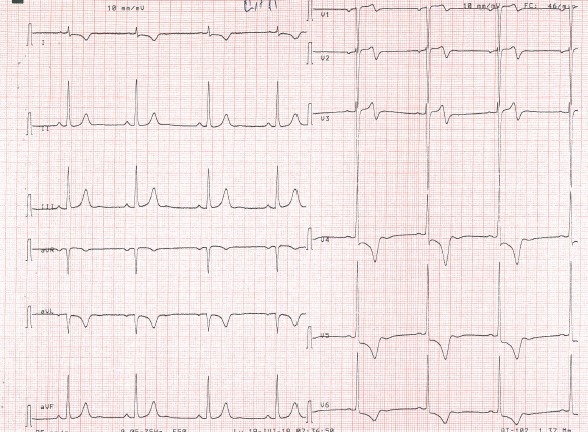
ECG showing negative T waves of epicardial ischemia of anterior topography

**Figure 2:**
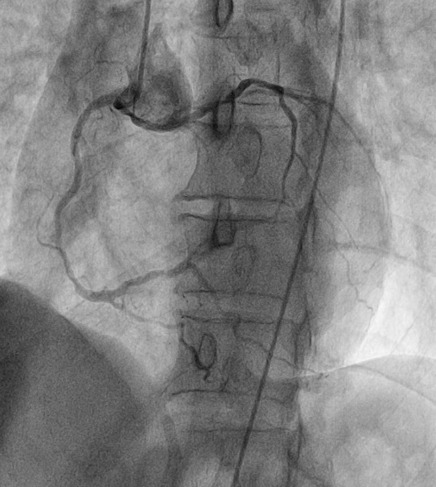
Left anterior oblique 30 View showing an ectopic connection of the left coronary artery with the right coronary artery

**Figure 3:**
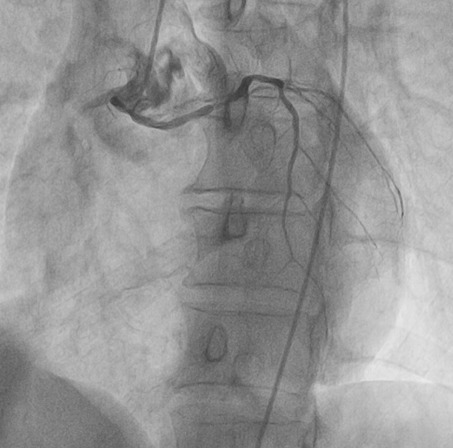
Significant stenosis (70-90%) of the middle segment of the left anterior descending artery

**Figure 4:**
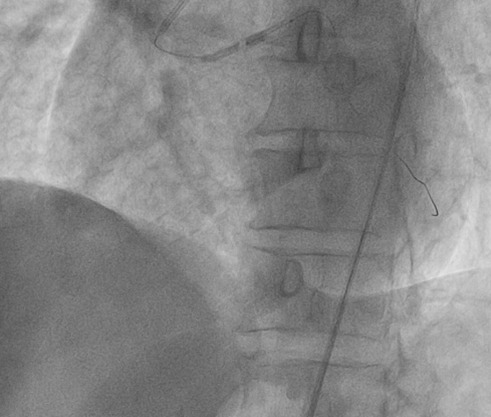
Angioplasty with placement of a 2.75 x 18mm active stent

**Figure 5:**
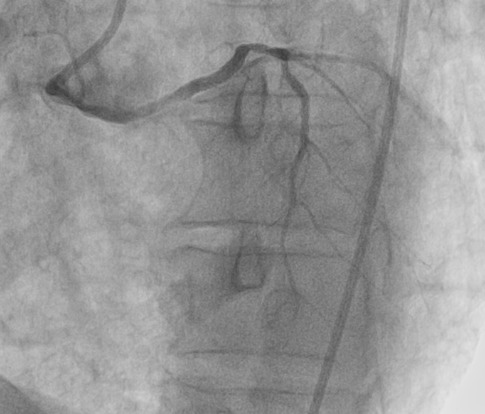
ECG showing negative T waves of epicardial ischemia of anterior topography

A 65 year old man having as cardiovascular risk factor chronic smoking, was admitted into our emergency room for acute high-risk non ST-segment myocardial infarction (NSTEMI). His physical examination noted crackling sounds up to half the two lungs, the rest of the physical examination showed no specific abnormalities. Rest ECG recorded a sinus rhythm at 50, with negative T waves of epicardial ischaemia in the anterior leads (Figure 1). Ultrasensitive troponin was high at 5953ng/l (normal range = 2-34ng/l). Transthoracic echocardiography showed a normal sized left ventricle hypokinetic the anterolateral, anterior and anteroseptal walls. The Left ventricle ejection fraction was estimated at 43% by the Simpson Biplan method. There was no mitro-aortic valve or pericardial involvement. Right femoral coronary angiography was performed. Catheterization of the left network could not be achieved despite the use of several types of diagnostic catheters. The JR4 6F catheter selectively opacified the right coronary artery that was moderately atheromatous without significant stenosis and then selectively opacified the left ectopic network that originates from the anterior right sinus (Figure 2). A middle sized left anterior descending artery had a significant 70-90% stenosis (culprit lesion) (Figure 3). An angioplasty (Figure 4) with placement of a 2.75x 18mm active stent was performed successfully (Figure 5). The patient did not benefit from intravascular ultrasonography (IVUS) or coronary scan for an accurate lesion assessment of this anomalous coronary connection as our center does not have these complementary examinations.

## Discussion

The angiographic prevalence of anomalous connections of coronary arteries is approximately 0.5-1% in adults free from congenital structural heart disease [1]. The diagnosis is more often made during the coronary angiography, but the initial course of the ectopic vessel remains sometimes uncertain. Connections in the opposite sinus raise technical difficulties as in the present case. In most cases, the ectopic ostium is located few millimeters from the normal position and sometimes even contiguous. It is then necessary to distinguish the connections with intramural course and without intramural course [2]. Angioplasty techniques for a coronary artery with an anomalous connection do not differ from the techniques used in case of an atheromatous lesion responsible for acute coronary syndrome on a normal coronary artery. The interventionist should not hesitate to introduce an additional 0.014 inch guide to secure a stable support whether for a connection in the contra lateral artery (a guide in the non-ANOCOR artery) or for a connection in the contra lateral sinus (a second guide in the ANOCOR artery). Caution should also be exercised when juxta-ostial or ostial stenting is performed when the ANOCOR ostium is very close to the ostium in the normal situation, although the direction of the two coronary arteries is at a low risk for the displacement of the atheromatous plaque in the non-ANOCOR artery. Non-invasive cardiac imaging (CT angiography and MRI) occupies a prominent place to specify the relationships with adjacent structures, mainly the aorta and the pulmonary artery. This imaging is particularly useful to help differentiate an inter-arterial course with poor prognosis from an intra septal course of favorable prognosis [3]. Intravascular ultrasonography is the imaging mode that makes it possible to affirm an intramural course that is a pejorative anatomical feature [4]. The sudden myocardial ischemia occurring especially during prolonged or repeated intense physical exertion is a probable mechanism in the occurrence of serious cardiac accidents. The risk of sudden death is particularly high in certain subgroups of patients: anomalous connection of the left coronary artery, presence of associated lesions (stenosis of the intramural segment, deformation of the coronary ostium) and young adult athletes subjected to particularly violent efforts [5]. The discovery of a proximal anomalous connection must lead to the determination of its potential severity. For benign anatomical forms, therapeutic abstention is the rule unless an associated atheromatous disease requires a revascularization. The American guidelines published in 2008 indicate surgery for left ANOCOR with an inter-arterial course as well as right ANOCOR with inter-arterial course if ischemia is documented. (Class I level of evidence B) [6]. an indication of class IIa is also recognized in case of coronary hypoplasia without documented ischemia.

## Conclusion

The occurrence of acute coronary syndrome can be problematic if the coronary artery responsible has an abnormal connection. Anatomical and radiological knowledge of major congenital coronary abnormalities and certain catheterization techniques should allow operators to avoid delaying beneficial myocardial reperfusion. Large, multicenter observational registers could provide a better understanding of the natural history of these abnormalities and provide more factual data for practitioners facing ANOCOR in adults.

## Competing interests

The authors declare no competing interests.

## References

[1] Hoffman JI (2014). Abnormal origins of the coronary arteries from the aortic root. Cardiol Young.

[2] Aubry P, Honton B, Leurent G, Halna du Fretay X, Dupouy P, Oug P, Juliard J-M (2014). Ectopic connection of the left coronary artery with the contralateral sinus with or without intramural pathway: how and why differentiating the. Ann CardiolAngeiol.

[3] Ropers D, Moshage W, Daniel WG, Jessl J, Gottwik M, Achenbach S (2001). Visualization of coronary artery anomalies and their anatomic course by contrast-enhanced electron beam tomography and three-dimensional reconstruction. Am J Cardiol.

[4] Angelini P, Flamm S (2007). Newer concepts for imaging anomalous aor-tic origin of the coronary arteries in adults. Cathet Cardiovasc Interv.

[5] Aubry P, HalnaduFretay X, Degrell P, Waldmann V, Karame N, Marijon E (2017). Sudden cardiac death and anomalous connections of the coronary arteries: What is known and what is unknown. Ann Cardiol Angeiol.

[6] Warnes C, Williams R, Bashore T, Child J, Connolly H, Dearani J (2008). ACC/AHA 2008 guidelines for the management of adults with congenital heart disease. J Am Coll Cardiol.

